# A legacy of progress in clinical pharmacology and social justice; remembering Peter Ian Folb

**DOI:** 10.2471/BLT.22.289501

**Published:** 2023-01-01

**Authors:** Karen I Barnes, Piero Olliaro, Melba F Gomes

**Affiliations:** a University of Cape Town Division of Clinical Pharmacology, Department of Medicine, Anzio Rd, Observatory, Cape Town, 7925, South Africa.; b Centre for Tropical Medicine and Global Health, Nuffield Department of Medicine, Oxford University, Oxford, England.; c School of Public Health and Community Medicine, Institute of Medicine, University of Gothenburg, Gothenburg, Sweden.

There are few effective and safe therapeutics for diseases that disproportionally affect impoverished populations, such as malaria and other neglected tropical diseases. But even these few therapeutics would not have been available without a critical mass in clinical pharmacology skills and a strong regulatory environment in the countries where these diseases are most prevalent.

The late Emeritus Professor Peter Ian Folb (1938–2022) was a visionary and a leading authority on the scientific basis of drug development, both as an academic and within medicines regulation. In 1996, he enabled the University of Cape Town Department of Pharmacology to become a World Health Organization (WHO) Collaborating Centre for Drug Policy and Research into Drug Development and Drug Safety, in conjunction with its sister department at the University of the Western Cape. This centre was the first WHO centre of its kind on the African continent and among low- and middle-income countries.

For 18 years, Folb was Chair of the South African Medicines Control Council.^1^ He advised on improvements to drug regulation and drug policy in 42 African and three European countries. He investigated and unravelled the apartheid government’s chemical and biological warfare programme for the South African Truth and Reconciliation Commission.^2^ Despite considerable political pressure, he led the Medicines Control Council’s principled stand of halting human trials on Virodene, a controversial drug based on a toxic industrial solvent that was claimed to be a treatment for human immunodeficiency virus, but that lacked sufficient evidence of efficacy or safety.^3^ He was awarded an honorary Doctor of Science degree by the University of Cape Town in 2016.

Beginning as a general practitioner serving an impoverished community in the rural town of Oudtshoorn in South Africa, Folb became a senior lecturer in clinical pharmacology at Guy’s Hospital, London (1966–1970) and eventually professor and head of the University of Cape Town Department of Clinical Pharmacology and chief specialist in internal medicine at Groote Schuur Hospital, Cape Town (1976–2003). Many of those who were influenced and guided by his vision continue to demonstrate the vital role that clinical pharmacology can play in the development and rational use of essential medicines to improve public health.^4^

Folb played a leading role in developing the national drug regulatory framework and essential drugs programme, which underpins the rational use of quality-assured medicines. He published several books on drug safety, and for many years was co-editor of *Meyler’s side effects of drugs* – the international encyclopaedia of adverse drug events.^5^^–^^7^

From 1993 onwards, Folb served on several international scientific committees.^8^ He was a member of the Drugs for Malaria committee, where he contributed to the scientific and strategic development of new treatments for malaria. He was also Chair of the WHO Special Programme for Research and Training in Tropical Diseases Special Task Force for Research into Severe Malaria. He held meetings with African regulatory authorities and directed the development of rectal artesunate as a pre-referral treatment of life-threatening severe malaria in infants and children in remote malaria-endemic settings where injectable treatment was not promptly available.^9^

From 1997 until his retirement, Folb was director of the South African Medical Research Council Traditional Medicines Research Programme. He led the development of integrated primary health-care guidelines for both traditional and allopathic health-care professionals and received two innovation awards from the Department of Science and Technology for the development of novel medicines from medicinal plants in southern Africa. His boundary-breaking work researching traditional medicines using rigorous scientific standards recognized and rewarded the intellectual property of the communities that identified medicinal plants.

In addition to his 1997–1999 work for the South African Truth and Reconciliation Commission, Folb made other important contributions to promote a more equitable society and redress past injustices. For example, he challenged the South African Medical and Dental Council about the death in police detention of anti-apartheid activist, Steve Biko.^10^

Folb balanced art and science, the personal and the professional. He wrote Haiku poetry, painted and created woodcuts, was a marathon runner, and an inveterate walker and a swimmer. He often said that when one walks fast for over 40 minutes, the brain switches to right-sided dominance. That is the time for solving problems and thinking strategically. 

Throughout his life, he exemplified the best that a committed clinician scientist with unwavering integrity can contribute to advancing knowledge, health, and challenging social injustice.
